# Early Versus Delayed Cranioplasty After Decompressive Craniectomy: A Systematic Review and Meta‐Analysis

**DOI:** 10.1002/brb3.71281

**Published:** 2026-03-10

**Authors:** Muhammad Taha, Yasir Saleem, Muhammad Waqar Shahid, Fatima Sajjad, Areesha Khan, Arsalan Khan, Muneeza Rizwan, Muhammad Farooq, Meriam Rafi Khan, Fahad Saleem, Mishal Imdad, Muhammad Sohaib, Israr Ahmad, Rizwan Ullah, Sana Ullah, Wajahat Hanif, Yashfeen Amjad, Nayab Mohsin, Mueed Iqbal, Abdullah Afridi, Fazia Khattak, Kamil Ahmad Kamil

**Affiliations:** ^1^ Khyber Medical College Peshawar Pakistan; ^2^ Loralai Medical College Loralai Balochistan Pakistan; ^3^ Ayub Medical College Abbottabad Pakistan; ^4^ Punjab Medical College Faisalabad Pakistan; ^5^ Gomal Medical College Dera Ismail Khan Pakistan; ^6^ Rehman Medical College Peshawar Pakistan; ^7^ Allama Iqbal Medical College Lahore Pakistan; ^8^ Internal Medicine Department Mirwais Regional Hospital Kandahar Afghanistan

**Keywords:** cranioplasty, decompressive craniectomy, timing, meta‐analysis

## Abstract

**Introduction:**

Decompressive craniectomy (DC) is a surgical procedure that involves the removal of a portion of the skull and is used to treat various conditions. However, this creates a significant defect in the skull that requires subsequent reconstruction through a procedure called cranioplasty. Cranioplasty aims to improve cerebral blood flow and enhance neurological functions. The timing of cranioplasty is still a topic of debate and can generally be classified into two categories: “early cranioplasty” and “delayed cranioplasty.” To provide evidence‐based recommendations for the optimal timing of cranioplasty, our meta‐analysis compares the safety and efficacy of early and delayed cranioplasty.

**Methodology:**

An electronic search was conducted on PubMed, Embase, and Cochrane. Multiple reviewers independently screened the studies using Rayyan software, and any conflicts were resolved through mutual discussion. Quality assessment was performed using the Newcastle–Ottawa Scale (NOS). Statistical analysis was carried out using Review Manager version 5.41, with *I* ^2^ statistics applied to evaluate heterogeneity.

**Results:**

Early cranioplasty was associated with increased risk of overall complications 1.43 (95% CI: 0.81 to 2.53; *p* = 0.22) and postoperative infections 1.36 (95% CI: 0.36 to 5.11; *p* = 0.65), shorter operative time −26.76 (95% CI: −41.20 to −12.32; *p* = 0.0003), and a reduced intraoperative blood loss −21.53 (95% CI: −38.30 to −4.77; *p* = 0.01). The neurological outcomes, that is, MMSE and GOS scores, favored the delayed cranioplasty or showed no significant difference, respectively.

**Conclusion:**

The meta‐analysis produced mixed results and required a prospective, multicenter, randomized controlled trial with specified outcome measures to establish a balance between surgical safety and functional recovery.

## Graphical abstract

• Early cranioplasty showed a higher (but non‐significant) risk

• RR 1.43 (95% CI: 0.81–2.53)

• Heterogeneity: *I* ^2^ = 57%

• Early cranioplasty associated with a non‐significant increase

• RR 1.36 (95% CI: 0.36–5.11)

• Early cranioplasty resulted in a significantly shorter duration

• MD –26.76 min (95% CI: –41.20 to –12.32)

• Heterogeneity: *I* ^2^ = 80%.

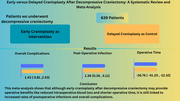

## 1 Introduction

Decompressive craniectomy (DC) is a well‐established surgical procedure used to treat malignant intracranial hypertension caused by conditions such as ischemic stroke, intracerebral hemorrhage, traumatic brain injury (TBI), and other acute brain trauma. By removing a portion of the skull, decompressive craniectomy can potentially save the lives of patients who do not respond to medical management by quickly reducing intracranial pressure and restoring cerebral perfusion (Honeybul and Ho, 2016; Gooch et al., 2009). However, this procedure results in a significant cranial defect, which necessitates subsequent reconstruction via cranioplasty (CP). Cranioplasty is performed to protect the brain and restore cranial contour and may even promote neurological recovery by improving cerebrospinal fluid dynamics and cerebral blood flow (Halani et al., 2017; Panwar et al., 2019).

Cranioplasty is associated with a considerable risk of postoperative complications, which depend on factors such as patient characteristics, surgical technique, and timing (Schuss et al., 2012; Shepetovsky et al., 2021). The optimal timing for cranioplasty after decompressive craniectomy remains a subject of ongoing debate. “Early cranioplasty” is generally defined as occurring within 2 to 3 months after the decompressive craniectomy, while “delayed cranioplasty” is performed after 3 to 6 months.

Proponents of early cranioplasty argue that it may reduce rehabilitation time, enhance motor function, and improve overall neurological recovery. They also highlight technical advantages, such as a shorter operative time due to less scar tissue formation (Piedra et al., 2014; Yadla et al., 2011). Conversely, supporters of delayed cranioplasty maintain that allowing sufficient time for the brain and surrounding tissues to recover may lower the risk of infection and complications related to wound healing. (Honeybul and Ho, 2016)

Recent studies on this topic have produced conflicting results. For instance, a comprehensive review and meta‐analysis by Malcolm et al. found that early cranioplasty was associated with improved motor outcomes; however, there was no significant difference in overall complication rates compared to delayed cranioplasty (Malcolm et al., 2016). Other cohort studies similarly suggested that while early cranioplasty led to a shorter operative time, the complication rates for both timing groups did not significantly differ (Schuss et al., 2012; Piedra et al., 2014).

Given the diversity in definitions, patient demographics, and outcome measures in the existing literature, a thorough synthesis of current findings is essential. This meta‐analysis aims to compare the safety and efficacy of early versus delayed cranioplasty following decompressive craniectomy, with a focus on postoperative complications, functional outcomes, and other clinically relevant endpoints, in order to support evidence‐based recommendations for optimal timing.

## 2 Methods

### 2.1 Study Design and Protocol Registration

This systematic review adhered to the guidelines set by the Cochrane Collaboration (Cochrane Handbook for Systematic Reviews of Interventions Cochrane Training 2025) and the Preferred Reporting Items for Systematic Reviews and Meta‐Analysis (PRISMA) framework (Page et al., 2021). It encompassed the study design, stepwise implementation, analysis, and presentation of findings. Additionally, the study protocol was registered in the International Prospective Register of Systematic Reviews (PROSPERO) under registration number CRD420251152263.

### 2.2 Data Sources and Search Strategy

The Preferred Reporting Items for Systematic Review and Meta‐Analyses (PRISMA) guidelines were followed to conduct this study. An electronic search was conducted across PubMed, Embase, and Cochrane from the time of their inception to June 29, 2025, using the keyword “Decompressive craniectomy” and the “clinical trial” and “observational study” filters were applied. The search string of this meta‐analysis was as follows;

(“Decompressive Craniectomy” [Mesh] OR “decompressive craniectom*” [tiab] OR “decompressive craniotom*” [tiab]) AND (“Cranioplasty” [Mesh] OR cranioplast* [tiab] OR “skull reconstruction” [tiab])

### 2.3 Study Selection and Eligibility Criteria

All studies identified through the online search were imported into the Rayyan software for screening, and duplicate records were removed. The remaining studies were initially screened based on their titles and abstracts. Full‐text articles were retrieved for further assessment if either reviewer found the abstract potentially relevant. Two independent reviewers (I. A. and A. K.) evaluated the eligibility of each study according to predefined inclusion criteria. Any disagreements were resolved through discussion and consultation with a third reviewer (A. K.).

Studies were included if they met the following criteria: (1) involved patients who underwent decompressive craniectomy; (2) early cranioplasty as Intervention (defined as ≤ 90 days after DC); (3) delayed cranioplasty as control (defined as > 90 days after DC); and (4) reported at least three relevant outcomes.

Exclusion criteria included (1) overlapping populations, defined by shared institutions and recruitment periods; (2) populations outside the scope of interest; (3) republished literature; (4) protocols without reported results; (5) reviews, abstracts, case reports, case series, background articles, expert opinions, or in vivo/in vitro studies; (6) duplicate data from the same clinical trial; or (7) absence of a comparator group.

In this review, “early cranioplasty” is defined as a procedure performed within 90 days (3 months) of the initial decompressive craniectomy. “Delayed cranioplasty” is defined as a procedure performed more than 90 days post‐craniectomy. This 3‐month threshold is the most commonly used delimiter in the contemporary literature. It aligns with the pathophysiological rationale that the initial period of brain swelling and metabolic instability largely resolves within this timeframe. At the same time, further delay may lead to increased scar tissue formation and anatomical challenges.

Randomized controlled trials (RCTs) were sought but are exceedingly rare for this surgical timing question. Therefore, observational cohort studies (both prospective and retrospective) and case series with comparator groups were considered for inclusion to comprehensively synthesize the available real‐world evidence, with their inherent limitations acknowledged and assessed.

### 2.4 Data Extraction and Outcomes

Two authors (M. R. K. and W. H.) extracted data from the included studies into an Excel sheet using a pre‐piloted form. Baseline data included recruitment period, intervention, control, follow‐up, time of cranioplasty after decompressive craniectomy, age, male population, and sample size. Outcomes were categorized into primary and secondary outcomes. The primary outcomes of this study included overall complications, postoperative infection, and operative time. The secondary outcomes were intraoperative blood loss, change in Mini‐Mental State Examination, and change in Glasgow Outcome Scale. See Supplementary Table S1for a detailed definition of outcomes.

### 2.5 Quality Assessment

We assessed the quality of observational studies using the Newcastle‐Ottawa Quality Assessment Form for Observational Studies (Ottawa Hospital Research Institute. n.d.), which includes three domains: selection, comparability, and outcome. We applied thresholds to convert the Newcastle‐Ottawa scores to AHRQ standards (good, fair, and poor). The selection domain was rated with a maximum of four stars, the comparability domain with a maximum of two stars, and the outcome domain with a maximum of three stars. Studies scoring 7–9 stars were rated as “low risk of bias,” studies scoring 5–6 stars were rated as “some concerns,” and studies scoring less than five stars were rated as “high risk of bias,” ensuring a comprehensive evaluation. This tool was also utilized in a previous study (Ali et al., 2025), ensuring methodological rigor.

### 2.6 Statistical Analysis

Review Manager 5.4 was used to perform statistical analysis. Treatment effects for binary outcomes were compared using a pooled risk ratio (RR) with 95% confidence intervals (CI), while continuous outcomes were analyzed using mean differences (MD) with 95% CI. The Cochran *Q* test and *I* ^2^ statistics were used to assess heterogeneity, with *P*‐values < 0.10 and *I* ^2^ > 50% considered indicative of significant heterogeneity (Higgins et al., 2003). The DerSimonian and Laird random‐effects model was applied to all outcomes (DerSimonian and Laird, 1986). A *p*‐value of <0.05 indicates statistical significance for clinical endpoints. The stability of the pooled estimates was assessed through a leave‐one‐out analysis, where each study was sequentially removed, and the remaining dataset was re‐analyzed to ensure that no single study unduly influenced the aggregated effect sizes and proportionately influenced the overall effect sizes.

## 3 Results

### 3.1 Searched Results

**FIGURE 1:**
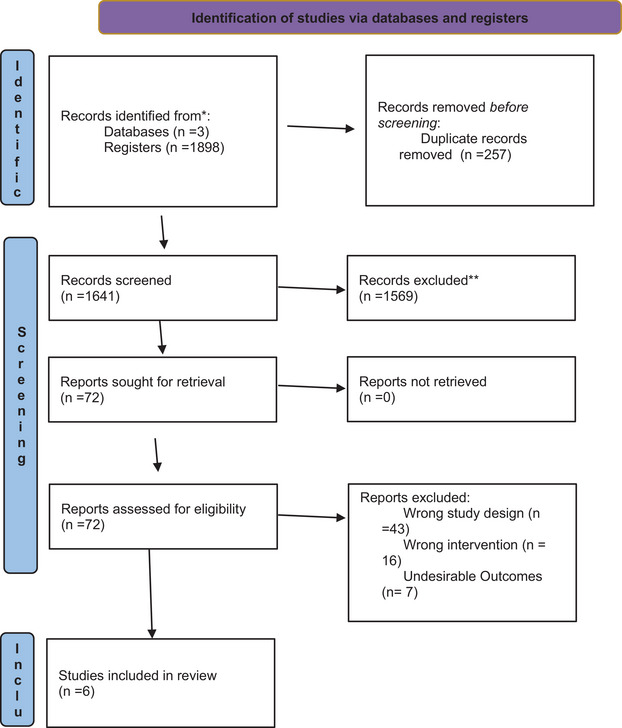
Prisma flowchart.

An initial search across three databases yielded a total of 1194 articles: 723 from PubMed, 348 from Cochrane, and 123 from Embase. This was achieved using a search string that included all relevant MeSH terms. After removing duplicates, 1037 articles remained for title and abstract screening. During the screening process, 994 articles were excluded, leaving 43 articles for eligibility assessment. Ultimately, six articles were included in our meta‐analysis (Goedemans et al., 2020; Sharma et al., 2024; Songara et al., 2016; Vreeburg et al., 2024; Yan et al., 2025; Zhao et al., 2025). Details of the screening process are illustrated in Figure **1**.

### 3.2 Study Characteristics

Our meta‐analysis included five observational cohort studies and one case series study involving a total of 639 patients, with 206 in the early cranioplasty group and 433 in the delayed cranioplasty group. The mean age ranged from 34.5 years to 44 ± 21.5 years in the early CP group and from 38.53 ±11.5 years to 44.6 ±14.1 years in the delayed CP group. The studies were published from 2016 to 2025 and were conducted in the Netherlands, India, Europe, Israel, and China. The indications for the initial decompressive craniectomy across studies included traumatic brain injury (TBI), ischemic stroke, intracerebral hemorrhage, and other causes, with mixed populations in most studies. The detailed baseline characteristics are mentioned in **Tables** **1** and **2**.

**TABLE 1:** Study characteristics

Author, year	Country	Recruitment Period	Type of study	Intervention	Control	Time of cranioplasty after decompressive craniectomy		Type of implant used	Follow‐up (months)
						Early CP	Late CP		
Goedemans 2020	Netherlands	October 2006– October 2018	Retrospective Case Series	Early Cranioplasty after supratentorial decompressive craniectomy	Delayed Cranioplasty after supratentorial decompressive craniectomy	≤ 3 months	> 3 months	Autologous bone flap and Alloplastic Implant	12
Sharma 2024	India	April 2017– April 2019	Prospective Observational	Early Cranioplasty (CP) after decompressive craniectomy	Delayed Cranioplasty (CP) after decompressive craniectomy	< 3 months	> 3 months	NR	3
Songara 2016	India	January 2012– December 2013	Prospective Observational	Early Cranioplasty after decompressive hemicraniectomy	Delayed Cranioplasty after decompressive hemicraniectomy	< 3 months	> 3 months	NR	1
Vreeberg 2024	Europe, Israel, Netherlands	2014 to 2020	Multicenter Prospective Observational Cohort	Early Cranioplasty following decompressive craniectomy (DC)	Delayed Cranioplasty following decompressive craniectomy (DC)	≤ 90 days	> 90 days	Autologous bone grafts and synthetic grafts	12
Yan 2025	China	January 2016– June 2023	Single‐center Retrospective Cohort	Early Cranioplasty (CP) following decompressive craniectomy	Delayed Cranioplasty (CP) following decompressive craniectomy	< 3 months	> 3 months	Polyetheretherketone (PEEK) and titanium implants	1
Zhao 2025	China	August 2017– December 2022.	Retrospective Cohort	Ultra‐early cranioplasty (CP)	Non‐ultra‐early CP	Very early, around 2–3 weeks	Much later, several months after DC	3D plasticized titanium mesh	4–53

**Abbreviations**: CP, Cranioplasty; NR, Not Reported.

**TABLE 2:** Patient characteristics.

Author, year	Age (years) Mean (SD)		Male (%)		Sample size	
	Early CP	Delayed CP	Early CP	Delayed CP	Early CP	Delayed CP
Goedemans 2020	43.4 (16)	44.6 (14)	35	50	37	108
Sharma 2024	40.63 (8.5)	38.53 (12)	NR	NR	30	30
Songara 2016	34.5	38.7	NR	NR	6	10
Vreeberg 2024	44 (22)	44 (24)	70	72	73	100
Yan 2025	57.5 (6.6)	57.1 (6.1)	54	47	37	49
Zhao 2025	44 (13.7)	45 (12.6)	61	69	23	136

**Abbreviations**: CP, Cranioplasty; NR, Not Reported.

### 3.3 Risk of Bias

According to the Newcastle‐Ottawa Scale, all of the included studies had a low risk of bias except Sharma et al. (2024), which had a moderate risk. The study's comparatively higher risk of Bias was attributable to a variety of factors, such as the presence of outcomes of interest at the outset (despite being a prospective cohort study), the absence of control over confounders other than age, sex, and marital status, and the lack of information regarding the follow‐up rates. A detailed assessment of the risk of bias is presented in **Supplementary Table S2**.

### 3.4 Outcomes

A total of six outcomes were assessed in this meta‐analysis, including Overall complications, Postoperative infection, Operative time, Intraoperative blood loss, Change in Mini‐Mental State Examination (MMSE), and Change in Glasgow Outcome Scale (GOS).

The outcomes were pre‐specified and categorized as follows: **Primary outcomes**: (1) Overall postoperative complications, (2) Postoperative surgical site infection. **Secondary outcomes**: (1) Operative time, (2) Intraoperative blood loss, (3) Change in Mini‐Mental State Examination (MMSE) score from pre‐CP to follow‐up, (4) Change in Glasgow Outcome Scale (GOS) score from pre‐CP to follow‐up.

#### 3.4.1 Primary Outcomes

##### 3.4.1.1 Overall Complications

**FIGURE 2:**
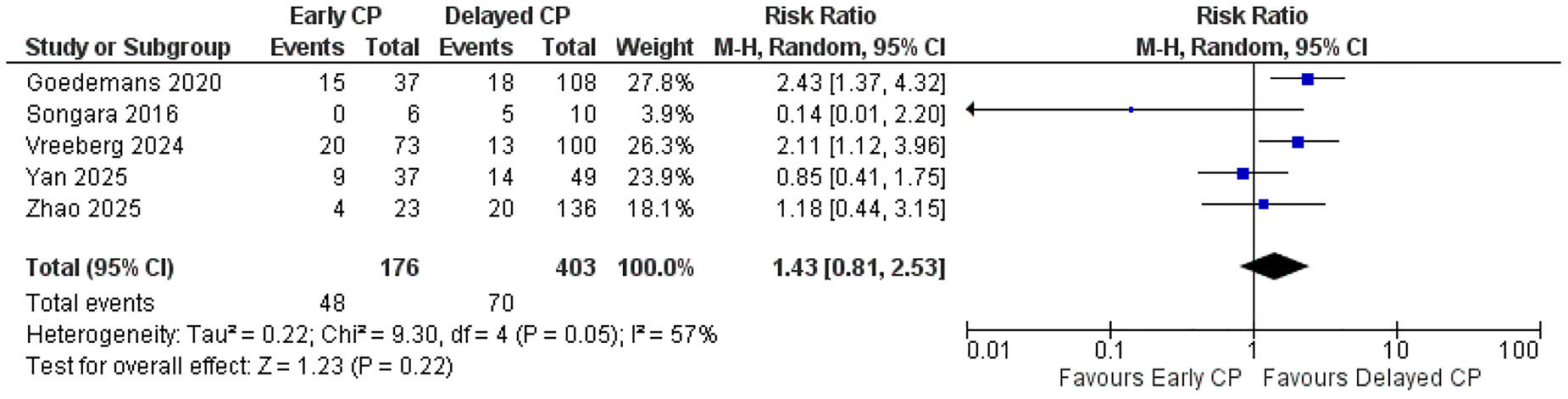
Forest plot of overall complications.

The overall complications were reported by all of the included studies except Sharma 2024. For this outcome, the total number of participants was 176 in the early CP group and 403 in the delayed CP group. The overall pooled estimate indicated that early CP was associated with an increased risk of overall complications, with an overall risk ratio of 1.43 (95% CI: 0.81 to 2.53; *p* = 0.22), but the result was not statistically significant (Figure **2**).

There was substantial heterogeneity among the studies (*I* ^2^ = 57%). A sensitivity analysis was performed, which indicated that after removing Yan 2025 and Songara 2016, the heterogeneity was reduced to 0% (**Supplementary Figure S1**). A subgroup analysis was performed based on the population being Asian or non‐Asian. The test for subgroup differences yielded a *p*‐value of 0.01, indicating that the type of population had a statistically significant impact on overall complications, suggesting that it might be a reason for the substantial heterogeneity (**Supplementary Figure S2**).

##### 3.4.1.2 Post‐operative Infection

**FIGURE 3:**
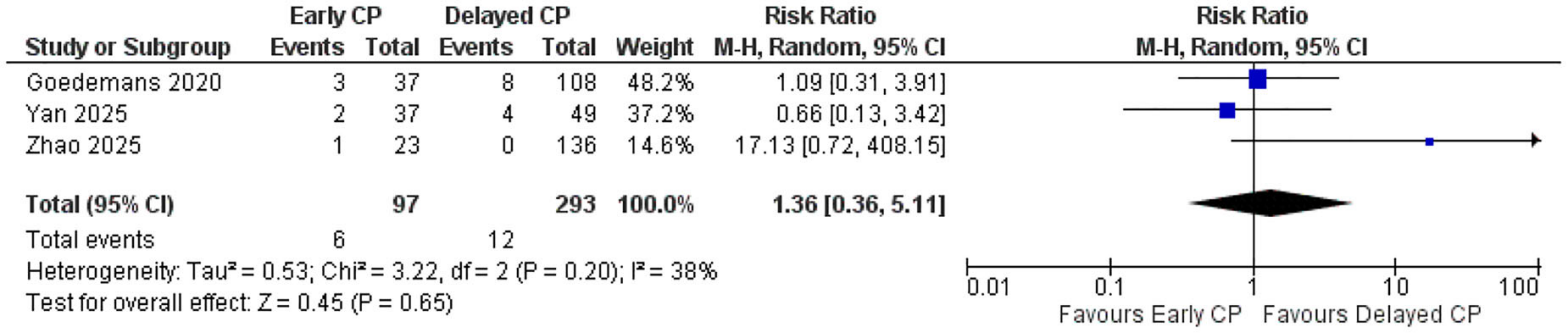
Forest plot of postoperative infection.

The postoperative infection was reported by three studies, including Goedemans 2020, Yan 2025, and Zhao 2025, with a total sample size of 97 participants in the early CP group and 293 participants in the delayed CP group. The overall pooled estimate of all three studies indicated that early CP had a statistically nonsignificantly increased risk of postoperative infection, with a risk ratio of 1.36 (95% CI: 0.36 to 5.11; *p* = 0.65) as compared to delayed CP. The heterogeneity among the studies was moderate (*I* ^2^ = 38%) (Figure **3**).

##### 3.4.1.3 Operative Time (mins)

**FIGURE 4:**
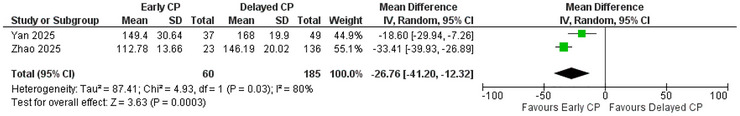
Forest plot of operative time.

Operative time was reported by only two studies, Yan 2025 and Zhao 2025, with a total sample size of 60 participants in the early CP group and 185 participants in the delayed CP group. The overall pooled estimate of the two studies favored early CP, as the mean difference between the early CP and delayed CP was −26.76 (95% CI: −41.20 to −12.32; *p* = 0.0003), indicating that early CP was associated with a statistically significant reduction in operative time. However, there was substantial heterogeneity between the two studies (*I* ^2^ = 80%) (Figure **4**).

#### 3.4.2 Secondary Outcomes

##### 3.4.2.1 Intraoperative Blood Loss (ml)

**FIGURE 5:**
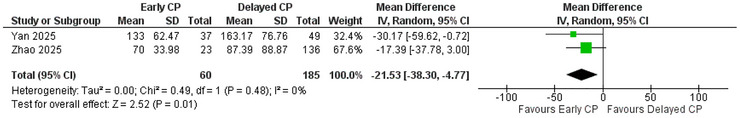
Forest plot of intraoperative blood loss.

Intraoperative blood loss was also reported by only two studies (Yan 2025 and Zhao 2025), with a total sample size of 60 participants in the early CP group and 185 participants in the delayed CP group. The overall pooled estimate of the two studies revealed a statistically significant mean difference in favor of early CP, as the mean difference between early and delayed CP was ‐21.53 (95% CI: −38.30 to −4.77; *p* = 0.01), suggesting that early CP was associated with a statistically significant reduction in intraoperative blood loss. The heterogeneity between the studies was non‐evident (*I* ^2^ = 0%) (Figure **5**).

##### 3.4.2.2 Change in Mini‐Mental State Examination

**FIGURE 6:**
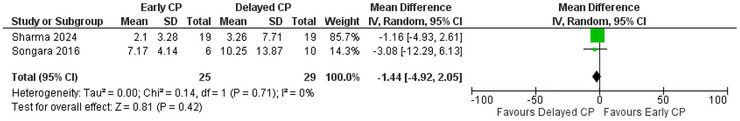
Forest plot of change in Mini‐Mental State Examination.

Only two studies reported the change in the Mini‐Mental State Examination, including Sharma 2024 and Songara 2016, with a total sample size of 25 participants in the early CP group and 29 participants in the delayed CP group. The overall pooled effect of the two studies favored the control over the intervention this time, with a mean difference of −1.44 (95% CI: −4.92 to 2.05; *p* = 0.42) between early and delayed CP, suggesting that early CP was associated with an overall decrease in MMSE. However, the p‐value suggested that the results were statistically nonsignificant. There was no heterogeneity between the included studies (*I* ^2^ = 0%) (Figure **6**).

##### 3.4.2.3 Change in Glasgow Outcome Scale

**FIGURE 7:**
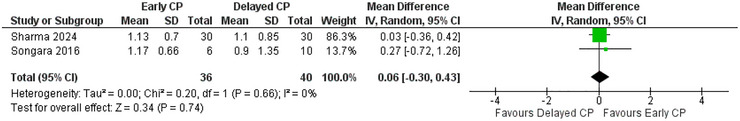
Forest plot of change in Glasgow Outcome Scale.

Change in Glasgow Outcome Scale (GOS) was again reported by just two studies, including Sharma 2024 and Songara 2016, with a total sample size of 36 participants in the early CP group and 40 participants in the delayed CP group. The overall pooled effect of both studies indicated no statistically significant difference between the early CP and delayed CP in terms of change in GOS, as the mean difference between the two groups was 0.06 (95% CI: −0.30 to 0.43; *p* = 0.74), suggesting that both groups had almost similar impacts on change in GOS. The heterogeneity between the studies was non‐existent (*I* ^2^ = 0%) (Figure **7**).

## 4 Discussion

There is considerable variation in the definition of “early” and “delayed cranioplasty.” Servadei et al. suggested that the timing of early cranioplasty can range anywhere from 4 to 16 weeks (Servadei and Iaccarino, 2015). Similarly, according to Piazza et al., early cranioplasty after 5 to 8 months might be associated with better surgical outcomes (Piazza and Grady, 2017). A systematic review by Xu et al. defined early cranioplasty as being performed anywhere from 1 to 3 months (2015). Another systematic review used the 3‐month time as a divider between early and late cranioplasty (Malcolm et al., 2016). A consensus statement from an international posttraumatic cranioplasty meeting agreed that ultra‐early cranioplasty will be defined as being performed at 6 weeks.

In contrast, early cranioplasty will be from 6 weeks to 3 months (Iaccarino et al., 2021). In conclusion, there is high evidence related to the fact that early cranioplasty is defined as being performed before 3 months following decompressive craniectomy, whereas delayed cranioplasty is performed beyond 3 months. In our systematic review, all of the included studies defined early cranioplasty as occurring either at or before 3 months. Furthermore, our analysis combined patients with different indications for DC (e.g., trauma vs. stroke). The pathophysiology, recovery trajectory, and risk profiles differ among these groups, which could confound the association between CP timing and outcomes. The included studies did not provide sufficient disaggregated data to perform an etiology‐based subgroup analysis. Future studies should report outcomes stratified by the etiology of the initial insult to allow for more nuanced recommendations.

The first outcome that was assessed in our meta‐analysis was overall complications. Regarding the overall complications, early CP was linked to a higher risk of overall complications, with a risk ratio of 1.43 (95% CI: 0.81 to 2.53; *p* = 0.22). This result was consistent with a case series performed by Tora et al., which reported statistically significantly higher odds of overall complication rates with early CP, with an odds ratio of 3.25 (95% CI: 1.73 to 6.09; *p* < 0.001) (2021). Zheng et al., however, reported no statistically significant difference in the overall complication rates between early and late CP in their meta‐analysis ([RR] 0.68 (95% CI: 0.46 to 1.29; *p* = 0.23) (2018). Another meta‐analysis by Chasles et al. also reported no significant difference in the odds of overall complication between the two groups ([OR] 1.02 (95% CI: 0.58 to 1.78; *p* = 0.95) (2025). Our result was statistically nonsignificant, and the heterogeneity was also substantial among the included studies (*I* ^2^ = 57%), suggesting that there might be some other factor responsible for the high risk. The subgroup analysis based on population revealed that the heterogeneity might be because there were two different populations being assessed in the cohorts. Songara 2016, Yan 2025, and Zhao 2025 included the Asian population, whereas Vree et al. 2024 and Goedemans 2020 included the non‐Asian population. The subgroup analysis yielded a *p*‐value of 0.01, indicating that the results significantly differed among the subgroups and might explain the heterogeneity. The subgroup analysis indicated that the non‐Asian population had a statistically significant increased risk of overall complications as compared to the Asian population, with a risk ratio of 2.28 (95% CI: 1.49 to 3.48; *p* = 0.0001) as compared to 0.88 (95% CI: 0.48 to 1.61; *p* = 0.67) for the Asian population. This finding was somewhat surprising because there is no clear evidence that suggests that ethnicity plays any role in determining the risk of complications following cranioplasty. There are, however, a limited number of studies, but they too report the risk being higher in the Asian population than in non‐Asian populations. A meta‐analysis comparing simultaneous cranioplasty and CSF shunt implantation with staged surgery, by Zhou et al., reported higher complications in the former group. A subgroup analysis based on ethnicity revealed that Asians were more prone to complications than other ethnic groups (2022). The higher risk of postoperative complications with early CP might have something to do with the fact that even after a mild cerebral trauma, the brain metabolism and CSF flow are significantly altered, and these derangements normalize slowly over a long period of time. Also, during this time, the brain is really vulnerable and might contain residual swelling. Thus, if a cranioplasty procedure is performed during this early period of brain vulnerability, it might lead to severe complications (Honeybul and Ho, 2016; Schuss et al., 2012).

Regarding the postoperative infection, early CP was again associated with a mildly increased risk of postoperative infection ([RR] 1.36 (95% CI: 0.36 to 5.11; *p* = 0.65). The result was statistically nonsignificant, however, with moderate heterogeneity (I^2^ = 38%). This is consistent with the normal trend in the literature. Morton et al., for example, reported a higher risk of postoperative infection in patients whose cranioplasty had been performed in <14 days after the initial craniectomy (p = 0.007) (2017). Similarly, a systematic review conducted by Yadla et al. also reported a mildly elevated risk of postoperative infection with early CP, with an odds ratio of 1.35 (95% CI: 0.53 to 3.1; *p* = 0.53) (2011). A retrospective cohort study by Im et al. also showed that late cranioplasty has a reduced hazard of causing postoperative infections as compared to early cranioplasty, with a hazard ratio of 0.502 (95% CI: 0.096 to 2.624; *p* = 0.414) (2012). Even though early CP increases the risk of postoperative infections, there are studies that favor early CP over delayed CP in terms of postoperative infection. For example, in a prospective cohort study conducted by Quah et al., there were no cases of postoperative infections in the early CP group.

In contrast, the delayed CP group had three cases of infection, though this result did not reach statistical significance (*p* = 0.55), making the authenticity of the result questionable (Quah et al., 2016). Another retrospective study by Oh et al. has reported a lower infection rate with early cranioplasty as compared to late cranioplasty (7% vs. 20%; *p* = 0.02) (2016). There are several reasons for the increased risk of infection with early cranioplasty surgery. As described earlier, after trauma, the brain becomes extremely vulnerable and needs time to heal completely. If, during this healing process, a cranioplasty is performed, then the chances of wound infection are significantly increased due to improper healing. Rosseto et al. described various other risk factors, including the presence of motor deficit, lower HB levels, and recent systemic infection, too, for the development of infection post‐cranioplasty (2015). It has also been suggested that the placement of a foreign material, that is, a cranial plate, also causes excessive inflammation leading to fluid accumulation that predisposes to infection (Thavarajah et al., 2012).

Operative time (mins) was one of the outcomes in which early CP was found to be superior to delayed CP. This is because the mean difference between the early CP group and delayed CP group was −26.76 (95% CI: −41.20 to −12.32; *p* = 0.0003), indicating a statistically significant reduction in operative time by early CP. The heterogeneity among the studies was substantial, however (*I* ^2^ = 80%), suggesting that the results could be biased because of any other factor. This result was in accordance with the general trend reported in the literature. A retrospective cohort study by Piedra et al. reported that the operative time of the early CP group was 23 min less than the delayed CP group (*p* = 0.0482) (2014). Xu et al. also reported a statistically significant and marked reduction in operative time in the early CP group as compared to the delayed CP, with a mean difference of ‐13.46 (95% CI: ‐21.26 to 5.67; *p* < 0.05) (2015). Another retrospective cohort study by Chun et al. reported a shorter operative time for early CP than delayed CP (95.33 mins and 133.67 mins, respectively, with a *p*‐value < 0.0001) (Chun and Yi, 2011). The reduction in intraoperative time with early cranioplasty has been attributed to the formation of less scar tissue, which enables simpler establishment of the dissection plane of the scalp flap while substituting the autologous bone piece (Sethi et al., 2022).

Early CP also outperformed delayed CP in terms of intraoperative blood loss (ml), as early CP was associated with a statistically significant reduction in intraoperative blood loss, mean difference: −21.53 (95% CI: −38.30 to −4.77; p = 0.01). The heterogeneity of this result was non‐existent (*I* ^2^ = 0%), indicating that the result was not biased. This result was also consistent with the available literature. For example, a prospective cohort study by Goyal et al. reported a statistically significant lower intraoperative blood loss with early CP as compared to late CP, with a mean difference of −137.3 mL (*p* < 0.05) (Goyal and Sharma, 2024). Chun et al. also reported a reduced estimated blood loss with early CP (336.67) as compared to late CP (573.3 mL) with a statistically significant *p*‐value < 0.0001 (Chun and Yi, 2011). Previous research indicates that delayed cranioplasty is linked to more extensive scarring, adherence of dura, and brain tissue retraction. This, in turn, makes surgical dissection really difficult, increasing the risk of dural tearing and leading to extensive blood loss (Goyal and Sharma, 2024).

Regarding the two neurological outcomes in our study, that is change in Mini Mental State Examination (MMSE) and change in Glasgow Outcome Scale (GOS), the early CP was surprisingly associated with a reduction in MMSE, suggesting that delayed CP is superior to early CP when it comes to MMSE (mean difference: −1.44 (95% CI: −4.92 to 2.05; *p* = 0.42). This result was contradictory to most results reported in the literature, which either favor early CP or show no significant difference. For example, Kim et al. reported that the gain score of K‐MMSE in the early group was higher (4.50±7.49) than that in the late group (−1.08±3.65, *p* = 0.019) (Kim et al., 2017). Kumar et al. also explained in their study that although there were insignificant differences in the MMSE scores of both groups, there was a significant improvement in the early CP group at all subsequent follow‐up durations (2018). Similarly, Ouyang et al. also reported in their study that at 6 months after the decompressive craniotomy, the MMSE scores of the early cranioplasty group were significantly higher than those in the late cranioplasty group (*p* = 0.040) (2020). In the case of GOS, however, no significant difference was observed between the two groups (mean difference: 0.06 (95% CI: −0.30 to 0.43; *p* = 0.74)). There was no heterogeneity among the included studies in both cases. This was in contradiction with the normal trend in literature, which usually reports an increase in the GOS score with early CP. Kumar et al. reported a greater improvement in GOSE scores in the patients assigned to the early CP group (2018). Ouyang et al. also reported a significantly greater improvement in GOS score in participants assigned to the early CP group as compared to delayed CP at 6 months follow‐up (0.041) (2020). Goyal et al. also reported a greater improvement in the GOS scores of the early group as compared to the late group, though the result could not reach statistical significance (Goyal and Sharma, 2024). These results of our study, apart from being statistically nonsignificant (*p* = 0.42 and 0.74), could also be biased because they were reported by just two of the included studies, and both of the studies were performed on the Asian population. As already explained, the Asian population might be more prone to overall complications due to a variety of factors (Zhou et al., 2022), suggesting that ethnicity might have something to do with the reduction in MMSE and no difference in GOS scores associated with early CP that is reported in our meta‐analysis. The improvement in neurological outcomes with early cranioplasty that is generally reported in the literature might have something to do with the increase in blood flow to the brain caused by cranioplasty. This is because cranioplasty protects the brain from the effects of atmospheric pressure that could otherwise cause a reduction in blood flow. Delaying cranioplasty leads to permanent scar formation that can compromise blood flow, causing reduced cortical function (Thavarajah et al., 2012). A key limitation of this analysis is the inclusion of predominantly observational studies. The decision for early or delayed CP is non‐random and heavily influenced by clinical factors such as persistent brain edema, infection risk, and patient stability, introducing significant selection bias and confounding by indication. While we employed rigorous quality assessment (NOS), the findings must be interpreted with caution. Our results underscore the critical need for well‐designed, multicenter RCTs to provide definitive evidence.

## 5 Conclusion

This meta‐analysis shows that although early cranioplasty after decompressive craniectomy may provide operative benefits like reduced intraoperative blood loss and shorter operative time, it is still linked to increased rates of postoperative infections and overall complications. Neurological outcomes are still mixed, and although the majority of research supports the early intervention, our findings do not clearly show an improvement in MMSE and GOS scores. Subgroup analyses indicated potential population‐based variations, indicating the need for more research on how ethnicity and patient‐specific characteristics affect results.

There is still disagreement on the best time to do cranioplasty due to variations in terminology, patient demographics, and research designs. To elucidate the balance between surgical safety and functional recovery, prospective, multicenter, randomized controlled trials with defined outcome measures are necessary. Patient selection, perioperative care, and geographical or ethnic differences that might affect the risk of complications should all get special consideration. To optimize cranioplasty time and enhance patient outcomes, evidence‐based standards must be established.

## Author Contributions

**Israr Ahmad** and **Arsalan Khan**: Methodology Software. **Areesha Khan**: Validation – **Meriam Rafi Khan**: and **Wajahat Hanif**: Formal analysis. **Mueed Iqbal**: and **Yasir Saleem**: Investigation. **Abdullah Afridi**: Resources. **Nayab Mohsin**: and **Yashfeen Amjad**: Data curation. **Sana Ullah**: Writing. Original Draft. **Rizwan Ullah, Muhammad Sohaib, Mishal Imdad, Fatima Sajjad, Muhammad Taha**: Writing – Review & Editing – **Fahad Saleem, Kamil Ahmad Kamil**: Visualization. **Muhammad Waqar Shahid**: Supervision. **Muhammad Farooq**: Project administration. **Muneeza Rizwan**: Funding acquisition.

## Funding

The authors have nothing to report.

## Ethics Statement

The authors have nothing to report.

## Conflicts of Interest

The authors declare no conflicts of interest.

## Supporting information




**Supplementary Material**: brb371281‐sup‐0001‐SuppMat.docx

## Data Availability

All data used are cited in the manuscript.
