# Supplementary material for: Early Versus Delayed Cranioplasty After Decompressive Craniectomy: A Systematic Review and Meta‐Analysis
Source: Brain Behav. 2026 Mar 10;16(3):e71281. doi: 10.1002/brb3.71281 (PMC12973139; doi:10.1002/brb3.71281)
Supplement: Supplementary file 1 — Supplementary Material: brb371281‐sup‐0001‐SuppMat.docx [file BRB3-16-e71281-s001.docx]

| **Primary Outcomes** | **Outcome** | **Definition** |
| --- | --- | --- |
| 1 | Overall Complications | The total number of adverse events or medical issues occurring as a result of cranioplasty surgery, including infections, hemorrhages, hydrocephalus, bone flap resorption, and other surgical or postoperative problems. |
| 2 | Post-operative Infection | The occurrence of any infection at the surgical site or within the cranial cavity following cranioplasty, confirmed by clinical signs or laboratory tests, requiring medical or surgical intervention. |
| 3 | Operative Time (mins) | The total duration of the cranioplasty procedure, measured in minutes from the first incision to the completion of wound closure. |
| **Secondary Outcomes** | **Outcome** | **Definitions** |
| 1 | Intraoperative Blood Loss (ml) | The volume of blood lost by the patient during the cranioplasty surgery, measured in milliliters. |
| 2 | Change in Mini Mental State Examination (MMSE) | The difference in cognitive function scores assessed by the MMSE test before and after cranioplasty, reflecting improvements or declines in areas such as memory, attention, language, and orientation. |
| 3 | Change in Glasgow Outcome Scale (GOS) | The difference in patient functional outcome measured by the Glasgow Outcome Scale before and after cranioplasty, indicating changes in neurological recovery and overall disability status. |

**Supplementary Table S1*:*** Definition of Outcomes

\

| **Study ID** | **SELECTION** | | | | **COMPARIBILITY** | | **OUTCOMES** | | | **TOTAL** |
| --- | --- | --- | --- | --- | --- | --- | --- | --- | --- | --- |
|  | S1 | S2 | S3 | S4 | C1 | C2 | O1 | O2 | O2 |  |
| Goedemans 2020 | * | * | * | * | * | * | * | * | - | 8* |
| Sharma 2024 | * | * | * | - | * | - | * | * |  | 6* |
| Songara 2016 | * | * | * | * | * | - | * | * | * | 8* |
| vreeberg 2024 | * | * | * |  | * | * | * | * | * | 8* |
| Yan 2025 | * | * | * |  | * | - | * | * | * | 7* |
| Zhao 2025 | * | * | * | * | * | - | * | * | * | 8* |

**Supplementary Table S2:** New Castle Ottawa Scale for Quality Assessment of Cohort Studies


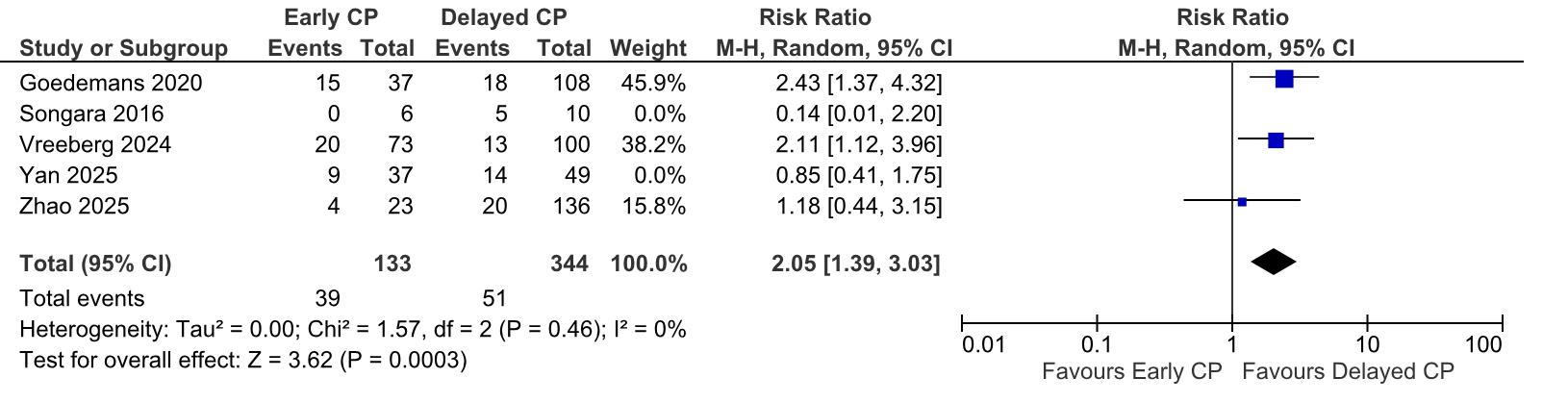


**Supplementary Figure S1:** Forest Plot of Overall Complications after Sensitivity Analysis


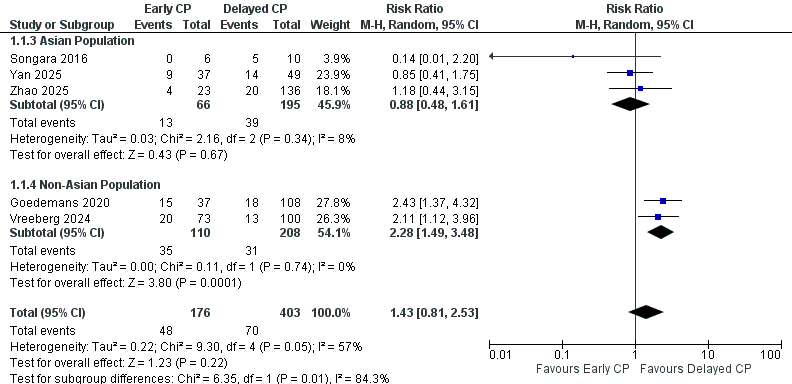


**Supplementary Figure S2:** Forest Plot of Subgroup Analysis of Overall Complications
